# Fungal Interactions Matter: *Tricholoma matsutake* Domination Affect Fungal Diversity and Function in Mountain Forest Soils

**DOI:** 10.3390/biology10101051

**Published:** 2021-10-15

**Authors:** Jie Zhou, Heng Gui, Shujiao Yang, Xuefei Yang, Lingling Shi

**Affiliations:** 1 Biogeochemistry of Agroecosystems, Department of Crop Science, Georg August University of Göttingen, 37075 Göttingen, Germany; jackzhou516@gmail.com (J.Z.); shilingling@mail.kib.ac.cn (L.S.); 2 Centre for Mountain Futures (CMF), Kunming Institute of Botany, Kunming 650201, China; guiheng@mail.kib.ac.cn; 3 Key Laboratory of Economic Plants and Biotechnology, Kunming Institute of Botany, Chinese Academy of Sciences, Heilongtan, Kunming 650201, China; lingling.shi@uni-goettingen.de; 4 Southeast Asia Biodiversity Research Institute, Chinese Academy of Sciences, Menglun, Mengla 666303, China; 5 Lijiang Forest Biodiversity National Observation and Research Station, Kunming Institute of Botany, Chinese Academy of Sciences, Lijiang 674100, China

**Keywords:** *Tricholoma matsutake*, shiro, fungal community, ectomycorrhizal fungi

## Abstract

**Simple Summary:**

Tricholoma matsutake forms a symbiotic association with hosts, developing mycelial aggregations, called ‘shiro’. The fungal diversity living in shiro soil play key roles in nutrient cycles for this economically important mushroom, but have not been profiled across large spatial and environmental gradients. Here, we mainly aimed to study the fungal characteristics in the bulk soil (non-shiro) and shiro, using phospholipid fatty acids and illuninan sequencing in five habitats across landscapes in southwestern China. T. matsutake causes a lower fungal diversity and simplifies the fungal community composition in shiro soil compared with non-shiro soil across five forest types. We also found that geo-graphical proximity was accompanied by a similar fungal community structure but different contributions from specific species under five forest types. Among the co-existed fungi, Oidiodendron were found to collaborate with T. matsutake, whereas Helotiales and Mortierella showed a negative correlation with it. The selection of these taxa may be related to their ability to compete with resources and different nutrient strategists in the soil. Overall, we conclude that T. matsutake generate a dominance effect to shape the fungal community and diversity in shiro soil across distinctive forest types.

**Abstract:**

*Tricholoma matsutake* forms a symbiotic association with coniferous trees, developing mycelial aggregations, called ‘shiro’, which are characterized by distinct chemical and physical properties from nearby forest bulk soil. The fungal diversity living in shiro soil play key roles in nutrient cycles for this economically important mushroom, but have not been profiled across large spatial and environmental gradients. Samples of shiro and non-shiro (nearby bulk soil) were taken from five field sites where sporocarps naturally formed. Phospholipid fatty acids (PLFA) and Illumina MiSeq sequencing were combined to identify fungal biomass and community structure. Matsutake dominated in the shiro, which had a significantly reduced saprotrophic fungi biomass compared to non-shiro soil. Fungal diversity was negatively correlated with the relative abundance of *T. matsutake* in the shiro soil. The fungal community in the shiro was characterized by similar fungal species composition in most samples regardless of forest types. Matsutake coexisted with a specific fungal community due to competition or nutrient interactions. *Oidiodendron* was positively correlated with the abundance of *T. matsutake*, commonly cohabitant in the shiro. In contrast, *Helotiales* and *Mortierella* were negatively correlated with *T. matsutake*, both of which commonly inhabit the non-shiro soil but do not occur in shiro soils. We conclude that *T. matsutake* generate a dominance effect to shape the fungal community and diversity in shiro soil across distinctive forest types.

## 1. Introduction

*Tricholoma matsutake* (S. Ito et Imai) is an ectomycorrhizal (ECM) fungi that forms symbiosis with *Pinaceae* and *Fagaceae* in regions such as China, Korea, and the Northern Hemisphere [1,2]. Matsutake can colonize plants in the rhizosphere, and develops a mycelial aggregation associated with their host roots and soil particles, named ‘shiro’ soil [1]. This unique and massive mycorrhizal mycelial aggregate generally forms 5–20 cm deep in mineral soil, which is visually distinct from the surrounding soil [3,4]. Fruiting bodies are formed in the shiro soil, where they can extract water and nutrients, therefore, a better understanding of the biotic and abiotic properties of shiro soil is crucial to *T. matsutake* production.

Interactions of microbial communities with *T. matsutake* in terrestrial environments have been widely studied, however, these studies have revealed divergent strategies [3,4,5,6]. For example, Lian et al. (2006) indicated that lower fungal diversity was found beneath the shiro in comparison to other zones (i.e., inside and outside the shiro) [3], which may be due to *T. matsutake* domination [7,8]. By contrast, Kim et al. (2013) found no difference in bacterial diversity between shiro and non-shiro soil [6]. Overall, *T. matsutake* seems to impart strong selection impacts on other microbial groups in the shiro soil.

*T. matsutake* domination generates unique physical and chemical properties of shiro soil, including a sandy texture, low pH and water holding capacity, which might indirectly affect the distinct soil microbial community [9,10]. The microbial groups in shiro soil may be important for the growth of *T. matsutake* [11], especially the existence of other soil fungi. Similar to *T. matsutake*, other mycorrhizal fungi play essential roles in carbon (C) and nutrient processes in terrestrial ecosystems [12]. However, *T. matsutake* commonly competes with other soil fungi for nutrients and living space [13,14], and then suppresses them with chemicals released from its hyphae, such as organic acids, lipids and special metabolites [15]. Conversely, some saprotrophic fungi may feed on mycorrhizal hyphae, consequently affecting its growth [14]. Therefore, future research on the interactions between soil fungal communities and *T. matsutake* in shiro soil are important for understanding how various fungi coexist with *T. matsutake*.

The soil microbial community is governed in part by the edaphic characteristic of the soil and aboveground plants, the regional climate conditions, and the interactions between these factors [16,17]. Importantly, plant types provide soils with abundant C and energy resources (i.e., rhizodepositions), thus exerting important and persistent effects on the formation of soil physic-chemical environments, and consequently selecting for specific soil microbial communities [18,19]. Here, we mainly aimed to study the fungal characteristics in the bulk soil (non-shiro) and shiro of *T. matsutake* combined, using phospholipid fatty acids (PLFA) and illuninan sequencing in variable habitats across landscapes in southwestern China. We hypothesized that: (1) *T. matsutake* has remarkable influences within the rhizosphere microbiome and shows different patterns between shiro and non-shiro soil due to the selection effect of *T. matsutake* [15]; (2) fungal community structure in the shiro dominated with *T. matsutake* across forest types show a similarity due to the domination effect of *T. matsutake*.

## 2. Methods and Materials

### 2.1. Site Description and Soil Sampling

Five annual *Tricholoma* produced forests were chosen in Yunnan, South-western China: Deqing (DQ1, DQ2), Lijing (LJ), Baoshan (BS) and Chuxiong (CX) (Table 1; Figure S1). *Tricholoma* are the major source of mushroom production in these areas. At each site, five random field replicate plots of 20 m × 20 m were set, which were separated with 25 m intervals. Here, we only set three field replicates in BS which was dominated with Pine. After removing the humus, soils were taken from the upper 10 cm in a fairy ring area (beneath the *Tricholoma*) on September 2015 with a soil drill during the mushroom harvesting season. In each of the five field replicates at each forest site, three sub-samples were mixed to form a homogeneous soil sample. The shiro areas at each forest site were 2 m away from each other. Another five sub-samples taken randomly inside each plot which were at least 5 m away from the shiro area were corrected as non-shiro soil. The front end of the shiro where the *Tricholoma matsutake* hyphae dominated was carefully selected according to morphological characteristics, where shiro has a significantly different sandy soil texture compared to the background soil. Once collected, samples were mixed, and any visible roots and stones manually removed. Soils were stored in sterile sealed bags on ice and then transported to laboratory to store under −20 °C. The relative abundance of genus *Tricholoma* in shiro soil was more than 40% of the whole community genotypes, significantly higher than that in the non-shiro soil (Figure S2). However, the relative abundance of *Tricholoma* varied with sites, with the highest relative abundance in DQ2 (>45%) and lowest in BS (20%). The composition of *Tricholoma* was simple, where 90% of the OTUs were classified as *Tricholoma* sp. Additionally, *Tricholoma* was the biggest ectomycorrhizal fungal group in this area (Table 1).

### 2.2. Soil and Plant Characterization

Total carbon (C) and total nitrogen (N) were determined by an elemental analysis (LECO CNS 2000, Leco Corporation, Joseph MI, USA). Soil pH was measured in suspension with a soil to water (m/w) ratio of 1:2.5 with a portable pH meter (HI 99121N, Hanna Instruments, Beijing, China), and total phosphorus (P) concentration was determined by ICP-MS analysis (Spectro, Kleve, Germany). Plant investigations were performed in each field plot, including plant species identification (species composition, diversity and ectomycorrhizal host species), shrub and grass coverage. Here, the dominant ectomycorrhizal host tree species were pine.

### 2.3. Phospholipid-Fatty Acid Analysis

Phospholipid fatty acids (PLFAs) were analyzed according to the protocol described by Frostegård et al. (1993) [19]. Briefly, 8 g of soil was extracted with a one-phase mixture of chloroform, methanol, and aqueous citric acid (1:2:0:8, *v*/*v*/*v*, pH 4.0) with two extraction steps. Phospholipids were separated from neutral lipids and glycolipids by solid phase extraction using an activated Silica gel (Silica gel Merck, particle size 0.063–0.200 mm). Then, phospholipids were analyzed with an Agilent 6890 gas chromatograph equipped with a flame ionization detector and an Ultra-2 column. The following PLFA biomarker were quantified into respective soil fungal groups: arbuscular mycorrhizal (AM) fungi was identified as 16:1ω5c, saprotrophic (SAP) fungi was identified by 18:2ω6, 9, and ectomycorrhizal (ECM) fungi was identified by the sum of 18:1ω9c, and 18:3ω6,9,12c [20].

### 2.4. DNA Amplicon and Illumina Sequencing of Fungal Communities

The primer ITS3_KYO2(5′-GATGAAGAACGYAGYRAA-3′) and ITS4-Barcodes (5′-TCCTCCGCTTATTGATATGC-3′) were used to amplify the ITS special region for mycorrhizal fungi. Amplifications were prepared using DNA (50 ng), as well as 0.5 mM each of forward and reverse primers in a 25 ul volume. PCR reactions were conducted under 94 °C for 5 min, then cycled 35 times under 94 °C for 30 s, followed by 30 s under 53 °C and 50 s under 72 °C, annealing three times under 53 °C, with a final elongation conducted under 72 °C for 5 min. PCR was purified with SanPrep DNA Gel Extraction Kit. After quantifying PCR with a Thermo Scientific™ NanoDrop™. PCR products were mixed homogeneously. The library was built using TruSeq^®^ DNA PCR-Free Sample Preparation Kit. After qualification by Qubit and qPCR, the DNA were sequenced using a v2 sequencing kit (2 × 250 bp) to perform Illumina sequencing on Miseq (high-though sequencing planform of Chengdu Institute of Biology, CAS).

The sequencing data were analysis by QIIME (v1.9.0). The noise reduction was performed using Denoiser 0.851 and Uchime, and chimeric sequences were detected using UCHIME and deleted. Sequences were shortened to 300 base pairs, and any sequences shorter than 300 base pairs were removed. Fungal sequences were independently clustered using USEARCH at 97% similarity. These sequences were then clustered into operation units (OTUs) using the UPARSE algorithm. The centroid sequence from each cluster is then run against either the USEARCH global alignment algorithm or against a database of high-quality sequences derived from the NCBI database. In total, 1395 OTUs were identified in all samples. The output was then analyzed using an internally developed python program that assigns taxonomic information to each sequence and then computes and writes the final analysis files. Based on the FUNGuild database 1.0, fungal OTUs were assigned to functional groups [21,22], which were performed at genus level, and only assignments with confidence levels of “highly probable” or “probable” were kept for further analyses. About 60% of the OTUs were matched to a functional guild in the FUNGuild database. The relative abundance of each functional group was similar with the sum of the relative abundance of all OTUs having a particular functional group.

### 2.5. Statistical Analysis

All the data sets were simplified to 6341 per sample by using the function “rarefy” in the R package vegan. Soil fungi richness analyses was performed by counting the OTU richness and standardized OTU richness with the function “diversity” and “scale” in the R package vegan, respectively. A Venn plot was created using ‘Calculate and draw custom Venn diagrams’ to differentiate between unique and shared OTUs dependent on feeding strategy. To evaluate the extent to which the individual OTU changes the way they interact with other OTUs in the network, significant co-presences or exclusions across the samples were identified by the CoNet method using a multiple ensemble correlation (guessing pair  =  50, row_minocc filter  =  10). Similarity measures were determined using Pearson and Spearman rank correlations. A co-occurrence network model was displayed by Cytoscape52, which indicated the interactions between fungal species. Nonmetric multidimensional scaling (NMDS) was performed to evaluate fungal community composition along the soil profile, as well as the correlation between fungal community structure and environmental factors (i.e., plant richness and diversity, as well as soil basic properties) based on the Bray-Curtis distances of the sequencing data using the vegan package. Two-way ANOVA was used to evaluate the effect of shiro and forest types on soil chemical properties. One-way ANOVA was used to evaluate the effect of shiro on specific microbial groups (ectomycorrhizal, saprotrophic and arbuscular mycorrhizal fungi biomass), fungi diversity and fungal richness with different trophic strategies across all forest sites, as well as the effect of forest types on plant diversity and richness. Permutational multivariate analysis of variance (PERMANOVA) was used to determine the effect of shiro and forest types on fungal community composition (OTUs). All statistical analyses were carried out with the R software v3.4.3.

## 3. Results

### 3.1. Characteristics of Soil in Tricholoma matsutake Fairy Ring

**Figure 1:**
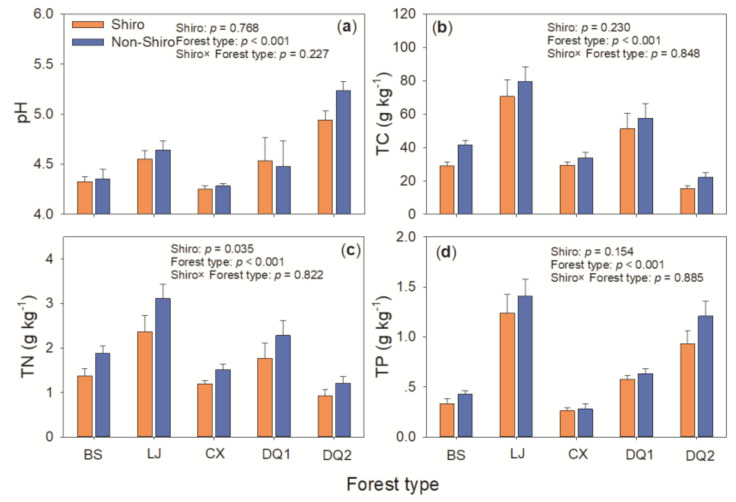
Soil pH (**a**), total carbon TC (**b**), total nitrogen TN (**c**), and total phosphorus TP (**d**) in the shiro and bulk soil from Deqing (DQ1, DQ2), Lijing (LJ), Baoshan (BS) and Chuxiong (CX) in Yunnan, South-western China.

*T. matsutake* fairy rings were generally down to a depth of 15 cm, with a remarkable sandy texture distinct from the soils outside the fairy ring. Soil pH, total C, N, and P varied with forest types (*p* < 0.01), but most of them showed generally similarity between shiro and non-shiro soils irrespective of forest types (*p* > 0.05; Figure 1). TN was 25% lower in shiro than that in non-shiro soil (1.54 vs. 1.93 g kg^−1^, *p* < 0.05), thus, it caused a higher C:N ratio in shiro compared with non-shiro soil (*p* < 0.05, Figure 1).

### 3.2. Fungal PLFA Biomass

**Figure 2:**
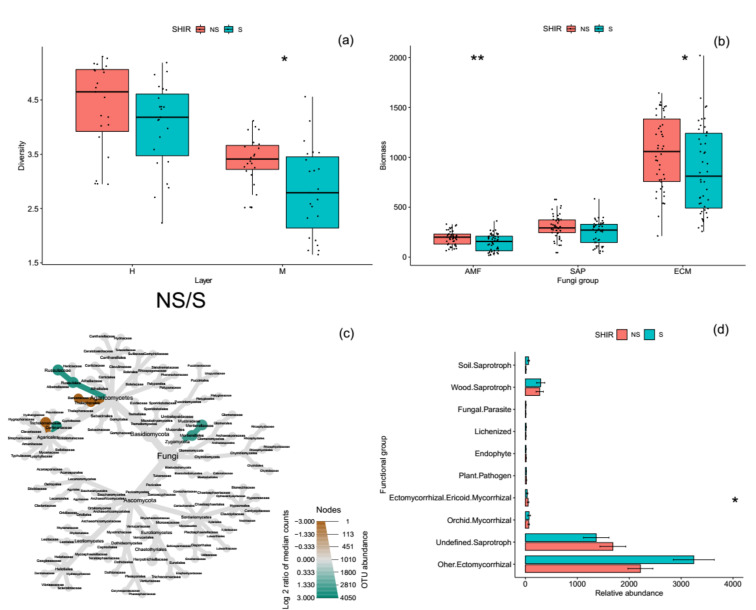
The changes of specific microbial groups based on, fungal diversity (**a**), phospholipid-fatty acid (**b**), fungi community composition (**c**), and their functions (**d**) in the shiro and non-shiro soil from all forest sites. * and ** indicate the significant differences between shiro and non-shiro soils at *p* < 0.05 and *p* < 0.01, respectively.

Microbial total PLFAs biomass were significantly higher in shiro than in non-shiro soil, due to an average 10% more fungal biomass in shiro (data not shown). Shiro soil contained 80% fungal biomass belonging to ECM fungi, with a lower biomass of AM fungi and SAP fungi (<10%). In terms of fungal group biomarkers, ECM and AM fungi were 180 and 450 nmol g^−1^ soil higher in the shiro compared with non-shiro soil, whereas the SAP fungi biomass was 120 nmol g^−1^ soil lower in shiro than in non-shiro soil across all forest sites (*p* < 0.05, Figure 2b).

### 3.3. Fungal Diversity, Species Composition and Functional Profiles

**Figure 3:**
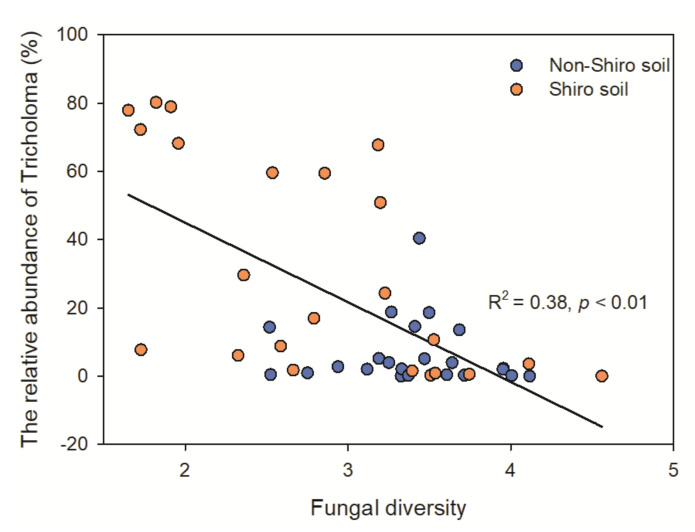
The correlation between fungal diversity and the relative abundance of *Tricholoma* both in the shiro and non-shiro soil from Deqing (DQ1, DQ2), Lijing (LJ), Baoshan (BS) and Chuxiong (CX).

The fungal diversity in shiro was 29% lower than that of non-shiro soil (1.4 vs. 1.8, Figure 2a), with lower fungal richness in shiro than in non-shiro soil, whereas there was no difference between forest types (*p* > 0.05, Table S2). The Pielou evenness index, indicating the distribution of each species in a community, indicated that shiro soil had a relatively uneven distribution compared with non-shiro soil (*p* < 0.001). The low fungal diversity results from shiro soil (the mycorrhizal zone for fruiting bodies) were considered due to the predominance of *T. matsutake* (Figure S2). Therefore, the farther away the forest soil fungal communities were from the fairy ring, the more diverse was their composition, indicating a negative correlation between fungal diversity and the relative abundance of *Tricholoma* observed (R^2^ = 0.63, Figure 3).

Members of the genera *Lactarius* dominated in the shiro soil, followed by *Russula*, *Amarita*, and *Boletopsis*, whereas the relative abundance of *Lyophyllum*, *Clayulina*, *Sarcodon*, and *Ramaria* were higher in the non-shiro soil compared with shiro (*p* < 0.05, Figure 2c). After classifying the taxa to fungal gilds, SAP fungi (e.g., wood and soil saprotroph, ecm-sap) fungi was 1 x lower in shiro compared to non-shiro soil (*p* < 0.05, Figure 2d). Similarly, other ECM fungi were decreased by 47% in shiro compared to non-shiro soil. In terms of trophic strategies of each fungal taxon, however, there were no differences in total fungi and SAP fungal species richness between shiro and non-shiro soil (*p* > 0.05, Table S3), whereas the richness of ECM fungi was 27% lower in shiro compared with non-shiro soil.

NMDS ordination of the fungal community did not completely separate shiro and non-shiro samples (Figure S3a), but samples from forest types tended to separate in different clusters along axis 1 (Figure S3b). Further, the fungal community structure in shiro soils was not only affected by soil properties (e.g., pH, TC, TN and TP), but was also affected by the diversity and richness of tree as well as its’ ECM host trees across different forest types (*p* < 0.05, Table S2).

### 3.4. The Correlation between Tricholoma and Other Soil Fungi in the Shiro Soil

**Figure 4:**
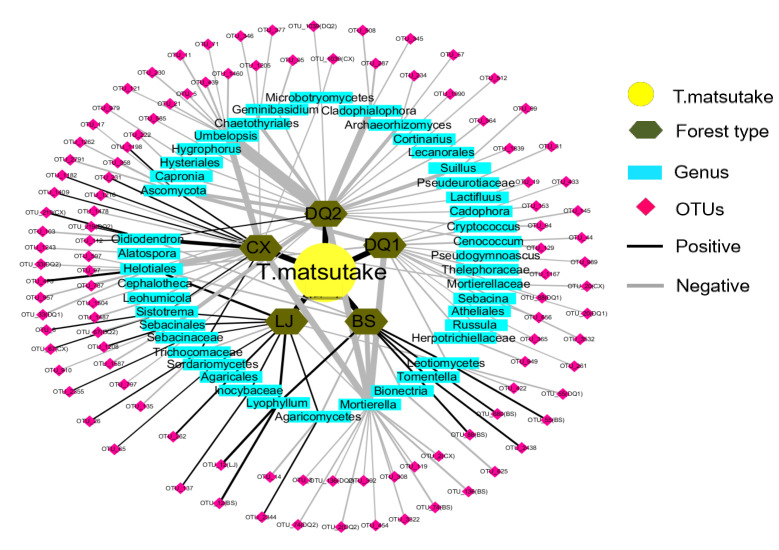
Correlation network of *T.matsutake* and other soil fungi in the shiro soil Figure 1. DQ2), Lijing (LJ), Baoshan (BS) and Chuxiong (CX) based on Spearman correlation analysis. The thickness of line indicates the R^2^ value.

In total, 78% of soil fungi was negatively correlated with *Tricholoma.* However, all the soil fungi were negatively correlated with *Tricholoma* in DQ1 and DQ2, whereas the positive correlation between soil fungi and *Tricholoma* was only found in BS, CX and LJ. At genus levels, however, there were similar soil fungi that correlate with *Tricholoma* across forest types, such as *Mortierella* and *Helotiales* which was negatively correlated with *Tricholoma*, whereas *Oidiodendron* was correlated with *Tricholoma* (Figure 4; Table 2).

## 4. Discussion

### 4.1. Variations of Fungal Community Differences between Shiro and Non-Shiro Soil

In the shiro soil, a significantly increased mycorrhizal fungal biomass (i.e., ECM and AM fungi) but reduced SAP fungal biomass compared with non-shiro soil was observed, which had some differences with previous studies. The increased mycorrhizal biomass was both due to ECM fruit body and AM fungi. AM fungi have their own C recourse from host roots [23,24], which might avoid competition with ECM fungi for C and energy. Given that AM fungi plays an essential role in environments with less nutrients (i.e., N and P) [25,26], the P limitation caused by *T. matsutake* immobilization may form an alternative explanation for the growth of AM fungi [27]. This was supported by the relative lower TP content in the shiro compared with non-shiro soil (Figure 1d). ECM fungi can capture nutrients released from decomposition by SAP fungi [28], and thus profit from their energy and C investment into enzyme activity. Furthermore, ECM fungi were found to release antibiotics that defend against saprotrophic activities under some conditions [29]. Therefore, the inhibition of growth and biomass of SAP fungi was observed in the shiro of *T. matsutake.*

To further investigate the fungal community composition, pyrosequencing was used, and a significant lower fungal diversity, as well as a lower ECM fungi richness, was observed (Figure 2), which was supported by previous studies [3,8]. Similar to other ECM fungi, *T. matsutake* can secrete antifungal substrates to inhibit other fungi, stimulating its own quantity and quality by decreasing competitors for nutrients and space [30]. This was further supported by the negative correlation between fungal diversity and the relative abundance of *T. matsutake* (Figure 3). On the other hand, soil properties were altered due to the uptake of nutrients or the exudation by *T. matsutake* in the shiro soil. In line with this, TN decreased in the shiro compared with non-shiro soil (Figure 1), and thus caused a shift in the fungal abundance and diversity between shiro and non-shiro soil (Figure 2; Table 3). Furthermore, host trees may indirectly shape soil fungal community composition because of their ability to alter the abiotic conditions in the surroundings [31]. Importantly, there was a significant relationship between the diversity of ECM colonized trees and fungal community structure (Table 3), due to their larger dependence on plant-microbe interactions as well as products of rhizodeposition [32].

As shown in the shiro soil, lower fungal diversity may be a common characteristic in ecosystems dominated with hyphae. For example, Voronina et al. (2011) and Halsey et al. (2016) observed a lower fungal diversity in the mycorrhizospheres of *Amanita* and *Laccaria* [33,34]. By contrast, Maŕi et al. (2020) found a higher fungal diversity in the fairy ring zone than in outside in grassland [35]. These inconsistent results may be explained by the idea that ecological and environmental conditions are the main driver for the variability in the microbial interactions within fairy rings [36]. This alteration of fungal diversity and simplification of soil fungal community composition in the shiro soil suggests that the hyphae of *T. matsutake* may shape a specific niche differently from the bulk soil.

### 4.2. T. matsutake Associated Fungal Community Similarities in the Shiro Soil across Geographic Locations

We found that fungal taxa interacting with *T. matsutake* were selectively enriched or reduced in the shiro in comparison to non-shiro soil. For example, members of the genus *Lactarius* became the largest group in the shiro soil, followed by *Russula.* These taxa were stimulated by the potential mycorrhizal-helper bacteria [37]. However, it was still unclear whether this alteration in the fungal community composition is related to the growth of *T. matsutake*. If the fungal community associated with *T. matsutake* in the shiro is unique and is shared through geographic locations, the *T. matsutake* domination may cause spatially distinct microbial community composition to be more similar. The fungal community structure in the shiro soil from five forest types in our case was similar (Figure S3), which indicates that soil fungi communities were shaped by the *T. matsutake*.

Although the species composition of fungi between forest types were similar, we found that forest types significantly changed the relative abundances of specific species. Different soils with five forest types sharing 55 fungal OTUs, potentially resulted in consistent fungal community composition, which may be attributed to the dominance of *T. matsutake*. Fungal species dissimilarity might be a result of distinctive plant communities. Considering that *Pinus yunnanensis*, *Quercus guyavefolia*, and *Pinus armandii Franch* were not classified as the same genus, they were expected to have a different genetic relationship in phylogenesis, which may form specific niches with physio-chemical properties caused by different litter quality and biomass, root exudates, and nutrient uptake ability [38,39]. This was indicated by the significant difference in pH, TC and TN between forest types (Figure 1; Table 3). For example, soil acidity seems to have a species-specific effect on the hyphal growth [40]. pH affects the growth capacity, mycelial density, colonization capabilities of mycorrhizal species [41]. Alteration in fungal performance under different pH influences their competitive abilities [42], affecting their abundance in the whole microbial communities. As a consequence, distinctive plant communities exert effects on soil fungal communities.

Our results also show that fungal species have distinct environmental requirements across five forest types, which is consistent with Shi et al. (2014) who suggested that soil fungal distribution is coincident with environmental pattern [43]. Among the five forest types, chemical sources and sinks (i.e., quality and quantity of litters, root exudates) differed to select spatial heterogeneity in which a variety of heterogeneous niches formed. In summary, we found a significant effect of *T. matsutake*, but in a limited area and also with interactions with other factors, such as local soil chemical properties and plant diversities.

### 4.3. Fungal Interaction Shape Fungal Community across Landscape

Interestingly, there were only 94 kinds of fungi, which accounted for 6.74% of the total fungi we found in this case, that were correlated with *T. matsutake*. This was in accordance with Bending et al. (2002) who found that most microorganisms had several lifestyles, such as accompanying or competing with ECM fungi under different environments [44]. Although *Mortierella* is a frequently reported genus associated with *T. matsutake* in the shiro, roots, and fruiting bodies [5,45], it was negatively correlated with the abundance of *T. matsutake*, which was accordance with Vaario et al. (2011) [4]. The exact mechanism of the suppression effect of *T. matsutake* on *Mortierella* may be that antibiotics secreted by fungal metabolites limit the ability of SAP fungi (i.e., *Mortierella*) as mentioned above [30]. Besides, the genus *Helotiales* was also negatively correlated with *T. matsutake*. Although *Helotiales* can form mycorrhizal association (i.e., ECM and ericoid fungi) with hosts to transfer C and nutrients [46], *T. matsutake* may colonize the root tips faster, which exhibited a priority effect and led to divergent communities because of the competition for limited root tips [47]. On the other hand, *Helotiales* sp. had diverse lifestyles, such as mycorrhizal-forming, ectomycorrhizal parasites, as well as saprobes, which was dependent on the local environmental conditions [48]. Thus, this may explain the decreased abundance of *Helotiales* in our case compared to the increased abundance in other studies.

However, the genus *Oidiodendron*, one kind of typical ECM fungi, collaborated with *T. matsutake* in five different forest soils. This was in accordance with Bending et al. (1997) who found that *Oidiodendron* could increase C and N availability in the rhizosphere by the stimulation of lignin degradation and litter decomposition [49], due to the increased exudation of cellulase and chitinase, consequently providing necessary nutrients that mitigate the nutritional deficiency of *T. matsutake* and facilitates its growth. Overall, the selection of these taxa may be related to competition for resources and space as well as their different nutrient strategies in the rhizosphere.

Information to interpret the mechanisms of the above noted dominance effect of *T. matsutake* on the associated fungal groups is still limited. The differential flow of C from ECM fungi to the other fungal groups may be a possible explanation for the multiple fungal performances. It has been extensively reported that energy-rich exudates from mycorrhizal fungi sustains the growth of other associated fungi [50]. However, plants can restrict the amount of C available to fungi according to the nutrient quantities they are receiving from it when co-colonized fungal species existed [51]. This would play a large role in the outcome of ECM competition growth because of the symbiotic interaction with hosts which are not C limited [47]. Besides C, *T. matsutake* may also exploit and immobilize minerals, thus causing a nutrient deficiency for coexisting microbial communities [27]. In addition to competition, associated fungal communities may also be influenced by mycotoxins such as cyanuric compounds or complex chemical compounds with phytotoxic and antimicrobial abilities produced by ECM mycelium [52]. This is consistent with previous studies who showed the negative effects of ECM fungi on other microbial communities [53]. For example, *T. matsutake* fruit bodies secrete hydrogen peroxide as a predominant inhibitor of other fungal growth [25]. Alternatively, the sandy soil that dominated in the shiro soil provides more oxygen and less moisture and nutrients than non-shiro soil, which may be an important selection tool for the associated fungal community [45,54].

## 5. Conclusions

Overall, our findings highlight that *T. matsutake* causes a lower fungal diversity and simplifies the fungal community composition in shiro soil compared with non-shiro soil across five forest types, which results from a direct selection effect of *T. matsutake* (e.g., competition for space and nutrients as indicated by lower TN). We also found that geographical proximity was accompanied by a similar fungal community structure but different contributions from specific species under five forest types, suggesting that the *T. matsutake* associated fungal communities were selected not only from the dominance of *T. matsutake* but also from the local soil properties and plant diversity. Among the fungi coexisting with *T. matsutake*, *Oidiodendron* were found to collaborate with *T. matsutake* in samples from all forest sites, whereas *Helotiales* and *Mortierella* showed a negative correlation with it. The selection of these taxa may be related to their ability to compete with resources and different nutrient strategists in the soil. Overall, our study suggests multiple symbiotic relationships between microbes and *T. matsutake*, which may form distinct fungal communities.

## Figures and Tables

**Table 1:** Site and stand characteristics of the study sites Deqing (DQ1, DQ2), Lijing (LJ), Baoshan (BS) and Chuxiong (CX).

Stand Parameters	DQ1	DQ2	LJ	BS	CX
Coordinates	28°05′ N, 99°37′ E	28°04′ N, 99°38′ E	27°00′ N, 100°10′ E	25°16′ N, 99°18′ E	25°10′ N, 101°00′ E
Altitude (m a.s.l)	3747	3452	3346	2452	2486
Annual mean temperature (°C)	7.3	7.3	9.0	17.3	17.6
Annual mean Precipitation (mm)	638	638	1275	600	1125
Tree species	*Quercus Semecarpifolia*	*Pinus densata*	*Pinus yunnanensis*, *Quercus guyavefolia*	*Pinus armandii Franch*, *Pinus yunnanensis*	*Pinus yunnanensis*

**Table 2:** The correlation between *T.matsutake* and other fungi groups. The + indicates positive correlation, whereas − indicates negative correlation.

	Mortierella −	Helotiales −	Oidiodendron +
BS	*Mortierella amoeboidea*	OTU_97	*Oidiodendron chlamydosporicum*
	*Mortierella humilis*		
CX	*Mortierella humilis*	OTU_957	*Oidiodendron griseum*
	*Mortierella cystojenkinii*	OTU_597	*Oidiodendron* sp *GK_2010*
	*OTU_119*	OTU_787	
DQ1	*Mortierella humilis*	OTU_33	
	*Mortierella* sp *WD32A*		
	*OTU_454*		
DQ2	*Mortierella amoeboidea*	OTU_33	*Oidiodendron griseum*
	*Mortierella humilis*		
LJ	OTU_3	OTU_475	

**Table 3:** Relationship of soil and plant variables with soil fungal community structure.

Variables	Shiro Soil				Non-Shiro Soil			
	NMDS1	NMDS2	R^2^	P	NMDS1	NMDS2	R^2^	P
pH	0.181	0.983	0.497	0.001	0.449	−0.893	0.067	0.241
TC	0.994	−0.112	0.274	0.001	0.199	0.979	0.326	0.002
TN	0.969	−0.245	0.282	0.001	0.163	0.986	0.297	0.003
TP	0.617	0.786	0.251	0.002	0.139	0.990	0.036	0.482
Tree_H	−0.434	−0.901	0.173	0.017	−0.074	0.997	0.001	0.665
Tree_S	−0.466	−0.885	0.188	0.012	0.600	−0.799	0.001	0.972
dbh	0.077	−0.997	0.003	0.950	−0.291	0.956	0.019	0.665
high	0.479	0.877	0.022	0.626	0.434	−0.901	0.008	0.803
Shrub_H	−0.269	−0.962	0.041	0.408	0.322	0.946	0.039	0.401
Shrub_S	−0.494	−0.869	0.054	0.832	0.009	0.999	0.046	0.337
Grass_H	−0.135	0.991	0.009	0.832	0.133	0.991	0.056	0.279
Grass_S	−0.017	0.999	0.108	0.076	−0.026	0.999	0.109	0.087
ECMtree_H	−0.319	−0.947	0.259	0.001	−0.981	0.195	0.001	0.978
ECMtre_S	−0.316	−0.948	0.335	0.002	−0.230	0.973	0.002	0.966

## Data Availability

The data in this study are readily available upon reasonable request to the corresponding author. BioSample accessions SAMN18710742 to SAMN18710833 have been published.

## Supplementary Materials

The following are available online at https://www.mdpi.com/article/10.3390/biology10101051/s1, Figure S1 Location of the study site. The circles indicate the position of Deqing (DQ1, DQ2), Lijing (LJ), Baoshan (BS) and Chuxiong (CX) in Yunnan, South-western China. Figure S2 The relative abundance of Tricholoma in Deqing (DQ1, DQ2), Lijing (LJ), Baoshan (BS) and Chuxiong (CX) in Yunnan, South-western China. Figure S3 Two-dimensional nonmetric multidimensional scaling (NMDS) ordination of fungal communities in shiro (S) and non-shiro (ns) soil in Deqing (DQ1, DQ2), Lijing (LJ), Baoshan (BS) and Chuxiong (CX) in Yunnan, South-western China. Figure S4 Venn diagram showing the specific and shared OTUs of shiro soil from Deqing (DQ1, DQ2), Lijing (LJ), Baoshan (BS) and Chuxiong (CX). Table S1 The tree diversity (Tree_H), richness (Tree-S), DBH (unit: m) and height (High, unit: m), and the diversity and richness of tree colonized by ectomycorrhizal fungi (ECMtree_H, ECMtree_S), as well as the diversity and richness of shrub and grass (Shrub_H, Shrub_S, Grass_H, Grass_S) in the Deqing (DQ1, DQ2), Lijing (LJ), Baoshan (BS) and Chuxiong (CX). One-way ANOVA was used to evaluate the effect of forest types on plant diversity and richness. Table S2 The effects of Shiro, forest type and their interactions on soil fungal richness, Fisher-α diversity and Pielou evenness. Table S3 Permutational analysis of variance (PERMANOVA) of total fungi and fungal trophic groups in all forest types.
