# Supplementary material for: Fungal Interactions Matter: Tricholoma matsutake Domination Affect Fungal Diversity and Function in Mountain Forest Soils
Source: Biology (Basel). 2021 Oct 15;10(10):1051. doi: 10.3390/biology10101051 (PMC8533266; doi:10.3390/biology10101051)
Supplement: Supplementary file 1 [file biology-10-01051-s001.zip › biology-1390203-supplementary.pdf]

Supplementary

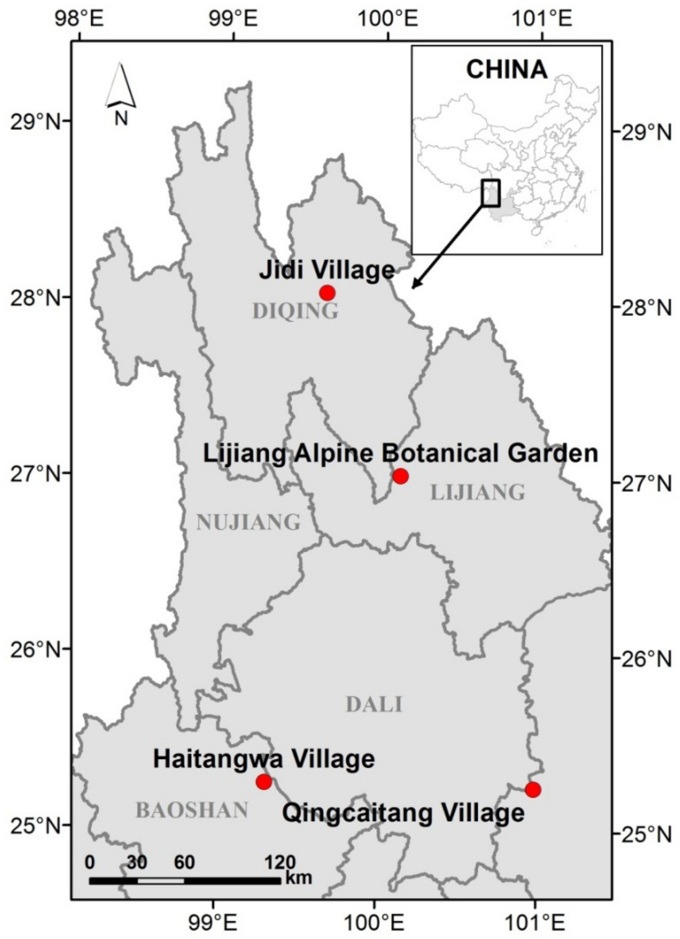

Figure S1 Location of the study site. The circles indicate the position of Deqing (DQ1, DQ2), Lijing (LJ), Baoshan (BS) and Chuxiong (CX) in Yunnan, South-western China.

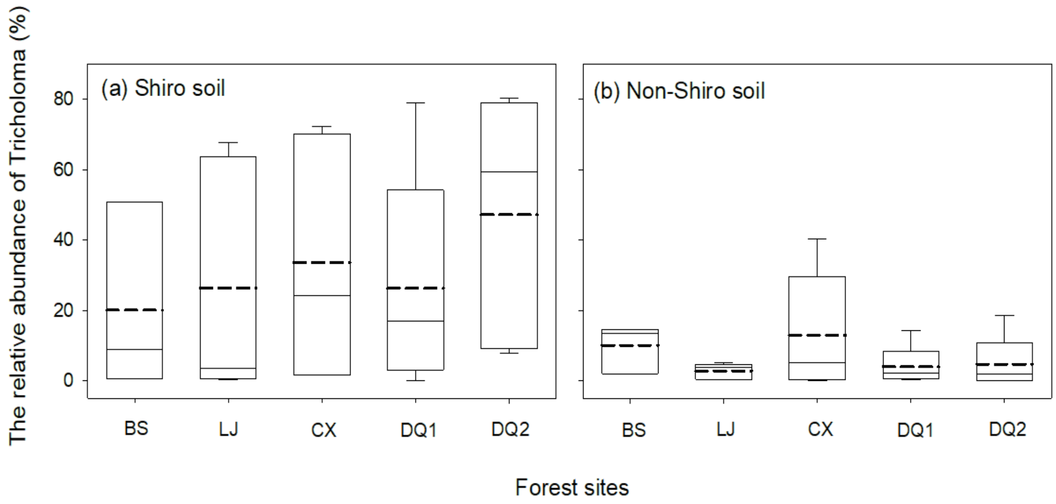

Figure S2 The relative abundance of *Tricholoma* in Deqing (DQ1, DQ2), Lijing (LJ), Baoshan (BS) and Chuxiong (CX) in Yunnan, South-western China.

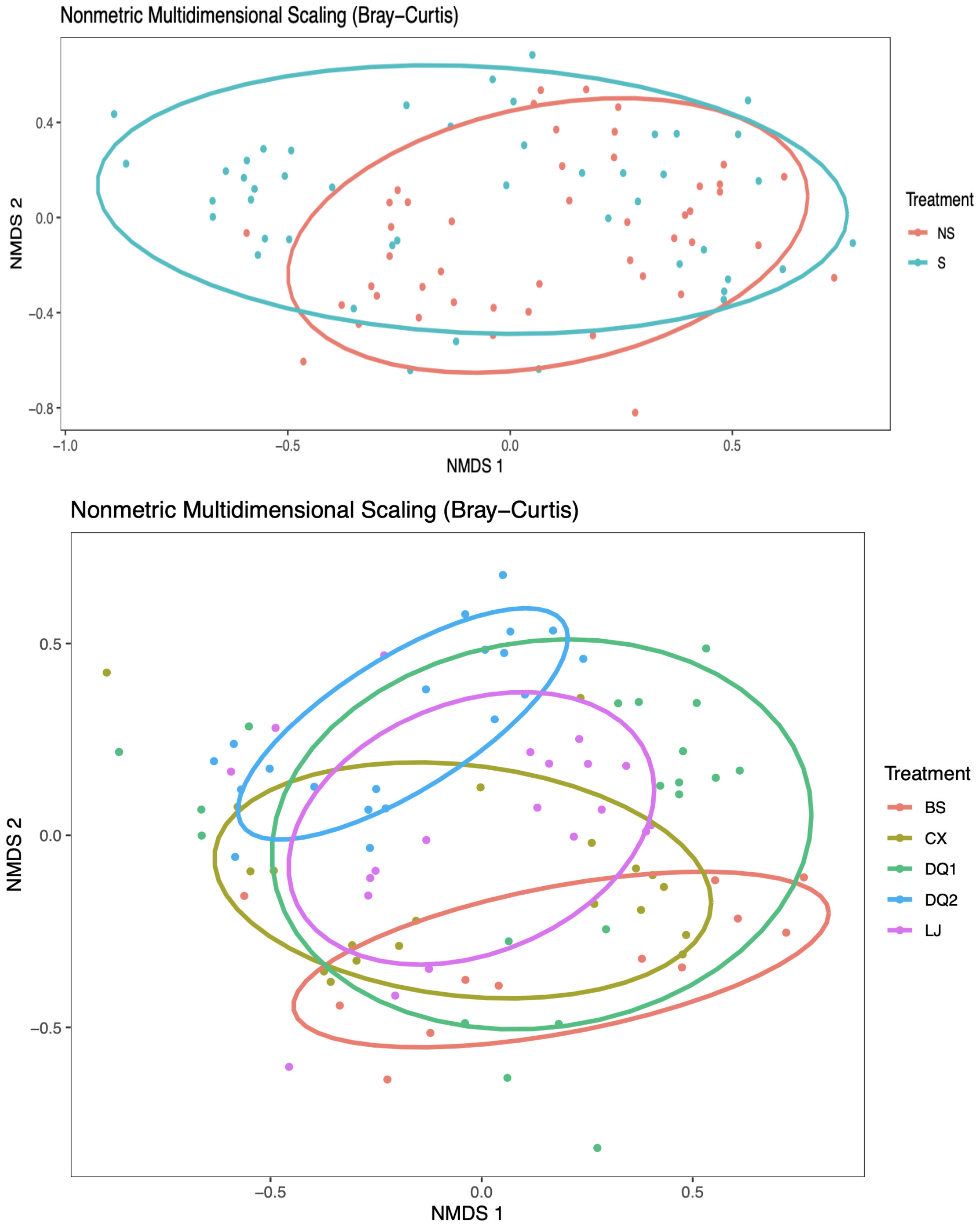

Figure S3. Two-dimensional nonmetric multidimensional scaling (NMDS) ordination of fungal communities in shiro (S) and non-shiro (ns) soil in Deqing (DQ1, DQ2), Lijing (LJ), Baoshan (BS) and Chuxiong (CX) in Yunnan, South-western China.

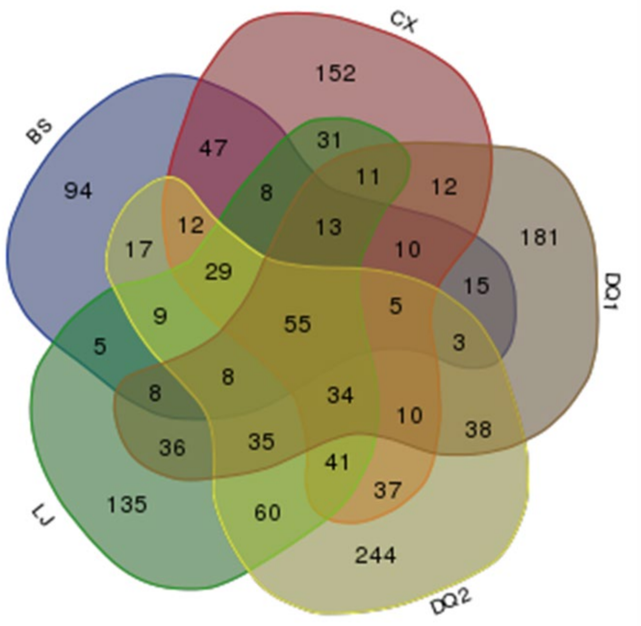

Figure S4 Venn diagram showing the specific and shared OTUs of shiro soil from Deqing (DQ1, DQ2), Lijing (LJ), Baoshan (BS) and Chuxiong (CX).

Table. S1 The tree diversity (Tree_H), richness (Tree-S), DBH (unit: m) and height (High, unit: m), and the diversity and richness of tree colonized by ectomycorrhizal fungi (ECMtree_H, ECMtree_S), as well as the diversity and richness of shrub and grass (Shrub_H, Shrub_S, Grass_H, Grass_S) in the Deqing (DQ1, DQ2), Lijing (LJ), Baoshan (BS) and Chuxiong (CX). One-way ANOVA was used to evaluate the effect of forest types on plant diversity and richness.

Variables	Forest type					
	BS	LJ	CX	DQ1	DQ2	P
Tree_H	1.291	1.115	1.549	1.290	0.603	0.001
Tree_S	2.158	1.838	2.309	2.022	0.957	0.001
DBH	4.444	4.234	6.354	6.562	5.476	0.031
High	4.223	3.308	3.640	4.657	4.787	0.001
ECMtree_H	1.253	1.046	1.471	1.074	0.571	0.001
ECMtree_S	1.990	1.601	2.073	1.593	0.913	0.001
Shrub_H	1.072	1.425	1.918	1.450	1.311	0.001
Shrub_S	1.365	1.973	2.295	1.894	1.545	0.001
Grass_H	0.449	1.536	2.024	0.938	1.790	0.001
Grass_S	0.536	2.296	2.516	1.982	2.496	0.001

Table. S2 The effects of Shiro, forest type and their interactions on soil fungal richness, Fisher-α diversity and Pielou evenness.

Variables	Shiro		Forest type		Shiro×Forest type	
	F	*p*	F	*p*	F	*p*
Richness	26.388	1.91e-^{5}***	1.646	0.191	1.305	0.292
Fisher-α diversity	25.048	2.74e^{-5}***	1.648	0.190	1.176	0.343
Pielou evenness	25.743	2.27e^{-5}***	0.459	0.765	0.780	0.548

Table. S3 Permutational analysis of variance (PERMANOVA) of total fungi and fungal trophic groups in all forest types.

	Shiro		Forest type		Shiro × Forest	
	R^2	P	R^2	P	R^2	P
Total fungi	0.086	0.001***	0.231	0.001***	0.081	0.135
Saprotrophic fungi	0.051	0.001***	0.275	0.001***	0.083	0.085
Ectomycorrhizal fungi	0.084	0.001***	0.184	0.001***	0.084	0.162
Plant pathogenic fungi	0.034	0.013*	0.236	0.001***	0.111	0.001***
Animal parasitic fungi	0.017	0.956	0.227	0.001***	0.110	0.757
Mycoparasitic fungi	0.061	0.051	0.373	0.001***	0.203	0.029*

* $p < 0.05$, ** $p < 0.01$, *** $p < 0.001$.
